# Damage–Safety Trade-Offs in the Transition of Apple Harvesting Methods: Impacts of Mechanized and Intelligent Harvesting on Fresh-Market Quality and Food Safety

**DOI:** 10.3390/foods15162787

**Published:** 2026-08-08

**Authors:** Yang Li, Hongjie Liu, Jianping Li, Pengfei Wang, Lixing Liu, Xin Yang

**Affiliations:** 1 College of Mechanical and Electrical Engineering, Hebei Agricultural University, Baoding 071000, China; 20242090211@pgs.hebau.edu.cn (Y.L.); liuhj@hebau.edu.cn (H.L.); jdwpf@hebau.edu.cn (P.W.); llxing@hebau.edu.cn (L.L.); yangxin@hebau.edu.cn (X.Y.); 2 Hebei Provincial Technology Innovation Center for Smart Agricultural Equipment, Baoding 071001, China

**Keywords:** apple, patulin, postharvest quality, food safety, damage risk index

## Abstract

Apple harvesting is transitioning from conventional manual picking to harvest-assist platforms, vibration-based mechanical systems, and selective robotic harvesting. For fresh-market apples, harvesting efficiency cannot be evaluated independently of mechanical damage, postharvest quality deterioration, and food-safety risks. Compression bruising, impact bruising, abrasion, cuts, punctures, and stem-end tearing can disrupt the peel, cuticle, and cellular structure to different degrees, triggering reactive oxygen species accumulation, membrane lipid peroxidation, cell-wall degradation, enzymatic browning, and increased respiration and ethylene metabolism. These responses may subsequently be amplified during storage, transportation, and processing, leading to softening, decay, shortened shelf life, and potential patulin contamination. Centered on the damage–safety trade-off, this review compares the technical characteristics and typical damage profiles of manual harvesting, vibration-based mechanical harvesting, harvest-assist platforms, and selective harvesting robots; explains how mechanical damage is transmitted from cellular physiological responses to food-quality and safety outcomes; and proposes conceptual frameworks for a Damage Risk Index and a Harvest Suitability Score. The central objective of intelligent apple harvesting should therefore be to improve efficiency while preserving appearance, texture, nutritional quality, storage stability, and food safety.

## 1. Introduction

Apples are among the most widely cultivated and economically valuable fresh fruits worldwide. According to data from the Food and Agriculture Organization of the United Nations, annual global apple production is approximately 86 million tonnes [1], with China contributing nearly 46% and ranking first in both cultivated area and output [2]. The commercial value of fresh-market apples depends on the coordinated expression of quality attributes such as external integrity, flesh texture, flavor, and storage stability. Any mechanical damage introduced during harvesting, however slight, may be progressively amplified along the subsequent supply chain, ultimately resulting in downgrading, increased decay, shortened shelf life, and even food-safety hazards [3].

Manual harvesting has long been regarded as the benchmark for protecting fresh-market apple quality because it combines high selectivity—fruit-by-fruit assessment of maturity—with flexible handling based on tactile feedback [4]. Nevertheless, the major apple-producing regions worldwide are facing an increasingly severe structural challenge. Apples bruise easily, and maturity varies markedly among cultivars and even among fruit within the same cultivar, making most tree-fruit mechanical harvesters excessively aggressive for fresh-market fruit [5]. To prevent bruising and ensure that fruit are harvested at optimum maturity, apples destined for the fresh market are still picked almost entirely by hand [6]. Apple picking is labor-intensive, physically demanding, and time-consuming. Labor is the largest single cost in apple production, and harvesting accounts for a substantial share of total production costs. As rural workers migrate to cities, rising wages, recruitment difficulties, and labor shortages have become major constraints on sustainable orchard development [7]. Meanwhile, the optimum apple harvest window is only 10–14 days, so the combined pressure of labor and time has made harvesting a critical bottleneck in industry upgrading.

These pressures have driven apple harvesting technology along four distinct pathways: conventional manual harvesting, vibration-based mechanical harvesting, harvest-assist platforms, and selective harvesting robots. Each pathway has advantages and disadvantages in efficiency, cost, and technological maturity; more importantly, they differ fundamentally in the types and severity of fruit damage and in their subsequent effects on postharvest quality and food safety [2]. Mechanized apple harvesting technologies can broadly be divided into semi-automated vibration systems, harvest-assist platforms, and fully autonomous robots [8]. However, while mechanical harvesters improve efficiency, they also cause fruit damage, which remains a major barrier to orchard mechanization. Collision-induced bruising is the most common form of mechanical damage during harvesting [9]. Although harvest-assist platforms are gradually being adopted, their overall penetration remains low; demonstrating their economic benefits and integrating additional functions, such as in-field sorting, could accelerate adoption [10].

The damage–safety trade-off is the central analytical framework proposed in this review. Its basic premise is that gains in harvesting efficiency should not be assessed independently of fruit damage, postharvest quality, and food safety. For systematic discussion, mechanical injuries are classified into compression bruises, impact bruises, cuts, punctures, stem-end injuries, and abrasions, and the concept of a damage profile is introduced to describe the composition of injury types produced by a specific harvesting method. Bruising is the most common postharvest mechanical injury and consists of subsurface tissue damage without peel rupture [11]. Bruises can occur during picking, storage, or transportation, causing quality loss and rejection by markets or consumers. They are not always immediately visible and can initiate a cascade of adverse effects, including cell disruption, tissue browning, decay, quality deterioration, and associated economic loss [12]. Different injury types compromise the peel barrier to different degrees and therefore differ fundamentally in their consequences for microbial invasion and food safety. Blue mold caused by *Penicillium expansum* is one of the most important postharvest diseases of apples and causes substantial economic losses through postharvest decay [13]. *P. expansum* is considered the principal fungal source of patulin contamination in pome fruits, particularly apples and apple products [14]. Patulin is the major mycotoxin of concern in apples and is produced mainly by *P. expansum* during fruit storage.

Several previous reviews have provided valuable engineering syntheses of apple-harvesting technologies. Ref. [15] reviewed the development and commercialization constraints of semi-automatic harvesters, harvesting robots, and harvest-assist platforms; Ref. [10] summarized technological progress in shake-and-catch systems, robots, and harvest-assist platforms for fresh-market apples; and Ref. [2] emphasized recent advances in automated equipment, perception, localization, and robotic harvesting. Their primary focus was system design, automation, operational performance, and commercialization. In contrast, the present review uses harvesting-induced damage as the connecting variable between engineering performance and food outcomes. It integrates method-specific injury profiles, cellular and physiological deterioration, postharvest quality, *Penicillium expansum*/patulin risk, and market-oriented decision support through the DRI/HSS framework and supply-chain control. The remainder of the review follows this logic. Section 2 summarizes the technological spectrum and typical damage profiles of the four harvesting approaches. Section 3 examines the cellular and physiological changes and quality-deterioration mechanisms induced by mechanical damage. Section 4 traces the progression from damage to food-safety risks, particularly *P. expansum* infection and patulin contamination. Section 5 presents a conceptual quantitative assessment framework. Section 6 proposes damage-mitigation and safety-control strategies covering preharvest management, low-damage equipment, in-field sorting, cold-chain management, and supply-chain traceability. Section 7 discusses international standards, regulations, and upstream HACCP control. Section 8 identifies research gaps and future directions, and Section 9 provides the conclusions.

This review does not attempt to exhaustively describe the engineering details of every harvesting system. Instead, it provides a selective, in-depth analysis from a food-science perspective, organized around the damage–safety pathway. This review employed a narrative review approach combined with a structured literature search. The databases searched included Web of Science, Scopus, PubMed, and China National Knowledge Infrastructure (CNKI). The search covered the period from January 2020 to June 2026, with backward citation tracing of included references to capture earlier seminal works. Search terms included “apple harvesting mechanization,” “apple mechanical damage,” “apple bruising,” “postharvest physiology apple,” “*Penicillium expansum* apple,” “patulin apple,” “robotic apple harvesting,” “harvest-assist platform,” and “vibration harvesting apple” in Boolean combinations. The inclusion criteria were: (1) peer-reviewed journal articles, conference proceedings, or official standards/regulatory documents; and (2) original studies reporting damage rates, postharvest quality changes, or food safety outcomes related to harvesting methods. The exclusion criteria were: (1) studies focusing solely on engineering design without quality-related.

## 2. Technological Spectrum of Apple Harvesting Methods

### 2.1. Manual Harvesting

Manual harvesting remains the predominant method for fresh-market apples [10,15]. Its principal advantages are selectivity and flexibility. Pickers can assess the maturity, coloration, and pest or disease status of each fruit and adjust grip force and angle in real time through tactile feedback, detaching the apple by twisting, lifting, or gently pulling [3,16]. For fresh-market apples that command premiums based on appearance and texture, no engineering alternative yet fully reproduces this level of fine manipulation [15].

Manual harvesting is not damage-free. Excessive gripping pressure, collisions among fruit in picking bags, and impacts when fruit are emptied into bins can all cause bruising [3,17,18]. These injuries are often latent bruises beneath an intact peel; they may be invisible at harvest but become evident during storage [19]. A further limitation is labor: harvesting can account for 20–35% of total apple production costs and is strongly affected by worker fatigue, experience, and the urgency of the harvest season [4]. In addition, non-picking activities such as moving ladders, climbing, and carrying fruit consume substantial working time [2,15], thereby limiting overall efficiency.

### 2.2. Vibration-Based Mechanical Harvesting

Vibration-based mechanical harvesting detaches fruit by shaking the trunk, scaffold limbs, or canopy and then collects the fruit using catching frames, cushioning pads, and conveyors [2,8]. This technology has considerable value for processing and cider apples: mechanically harvested ‘Brown Snout’ cider apples, for example, require only 22% of the labor cost of hand harvesting [20]. For fresh-market apples, however, the complexity and severity of the resulting damage profile remain fundamental obstacles [8].

During detachment, fruit may experience multiple mechanical loads, including microstructural disruption from forced vibration, impact bruising during the fall, stem-end tearing, cuts or punctures caused by broken branches, and repeated collisions during conveying [3,8,21]. Studies indicate that even with optimized cushioning, only approximately 85% of fruit harvested under the best vibration conditions meet the highest USDA grade [8,22], meaning that 15% are downgraded because of damage. Although cushioning materials can markedly improve impact tolerance [23], vibration harvesting still cannot consistently satisfy the quality requirements of premium fresh markets [8,10,15] and is more realistically positioned for processing fruit [20].

### 2.3. Harvest-Assist Platforms

Harvest-assist platforms use self-propelled mobile structures to position workers at an appropriate picking height and often integrate conveyors, bin positioning, and in-field grading to reduce non-picking labor such as ladder movement and climbing [10,15]. Because workers retain responsibility for judging maturity and appearance, this approach is widely considered the intermediate pathway with the greatest near-term potential for commercial adoption.

Damage risks on these platforms are concentrated in conveying and bin filling, where fruit may undergo repeated collisions while changing direction, being elevated, or dropping into bins [15,24]. Optimizing distributor design—for example, replacing a multi-opening distributor with a conical distributor—can keep bruising within fresh-market limits while doubling picking efficiency [24]. More advanced platforms integrate in-field sorting and have achieved harvest-induced bruise downgrade rates as low as 0.4% [25]. Compared with fully autonomous robots, harvest-assist platforms are technologically mature and relatively low-risk, making them an important pathway for mechanizing fresh-market apple harvesting in the short to medium term [10,15,24].

### 2.4. Selective Harvesting Robots

Selective harvesting robots comprise a perception system, robotic arm, end-effector, and mobile platform. Machine vision identifies fruit position and maturity, after which the end-effector grips, twists, or uses suction to detach the fruit [5,6,26]. Recent advances in deep learning, three-dimensional vision, and soft robotics have substantially improved picking success and reduced damage [2,27].

Recent field trials have shown that a dual-arm apple-harvesting robot can achieve an approximately 80% first-attempt picking success rate, with 91% of harvested fruit retaining the highest USDA grade and bruise rates of 2.4–4.9% [28,29]. In field tests of a vacuum-based soft end-effector, all tested apples met the Extra Fancy grade [30]. Nevertheless, robotic harvesting still faces severe occlusion, variable illumination, positioning errors, insufficient speed (6–8 s per fruit, substantially slower than manual picking), and high equipment costs [10,27]. Orchard architecture also strongly affects success: rates of 82.4% have been reported in well-pruned young orchards but only 65.2% in older orchards [30].

**Figure 1:**
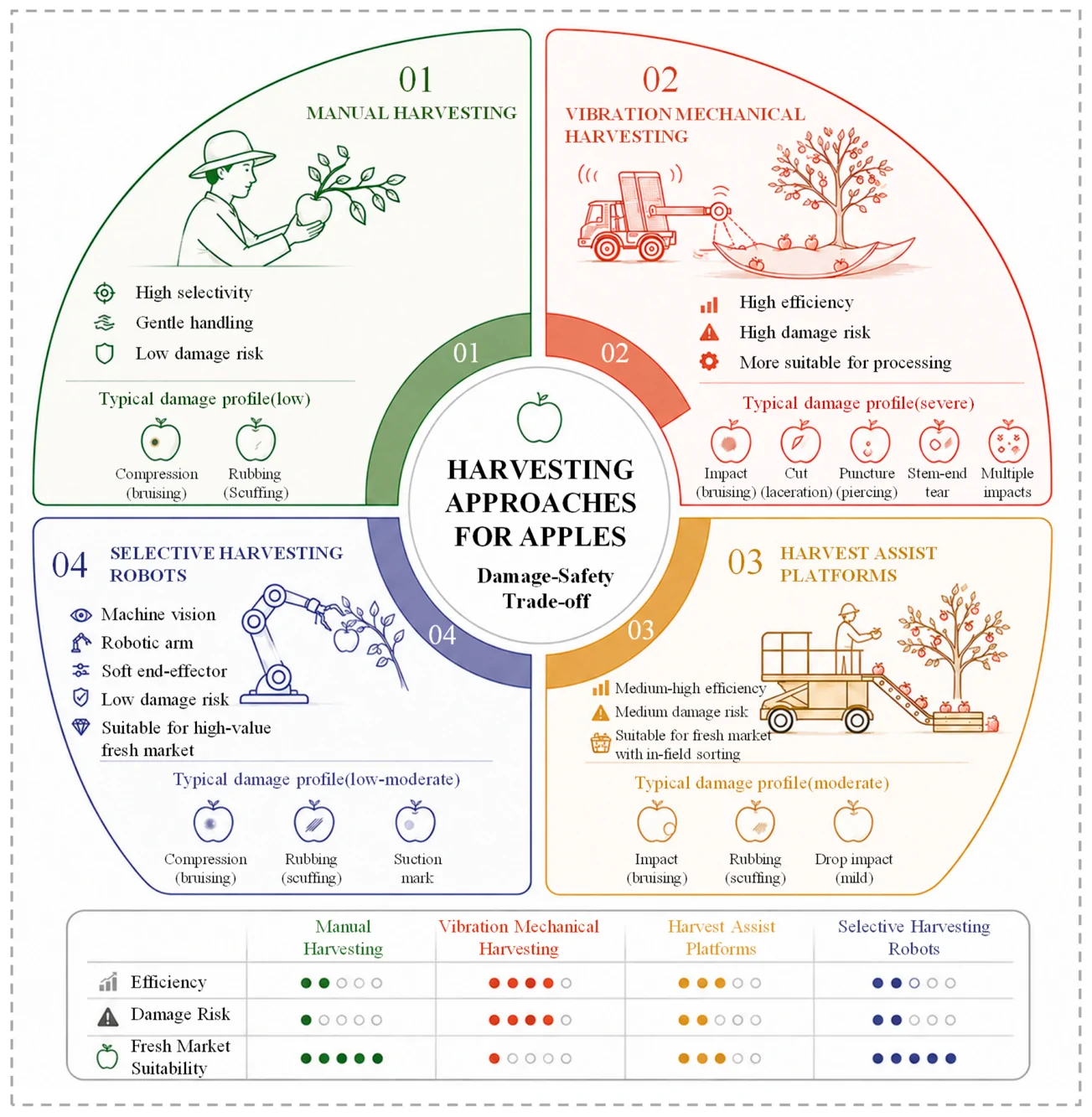
Comparison of apple harvesting methods and their typical damage profiles. Solid dots represent the rating score, with a maximum of five dots. More solid dots indicate higher efficiency and better fresh market suitability. For the damage risk indicator, more solid dots correspond to a greater risk of fruit damage.

From a damage perspective, the principal injuries associated with robotic harvesting are compression bruises caused by end-effector pressure, suction marks, abrasion caused by slippage, and collisions [17,25,31]. Soft end-effectors distribute gripping pressure through conformal contact and can reduce surface bruising and tissue crushing [31]. However, most robotic harvesting studies still use picking success as the primary performance metric, while systematic evaluation of postharvest storage quality remains limited [10,15]. The four harvesting methods emphasize different combinations of injury types; their typical damage profiles are compared in Figure 1.

### 2.5. Quantitative Synthesis of Representative Studies

To convert part of the narrative evidence into an accessible comparative record, Table 1 summarizes representative quantitative findings reported in studies cited in Section 2 and Section 6. The selected outcomes include labor or production costs, highest-grade retention, fruit downgrading, bruise incidence, robotic picking performance, and representative damage-control and detection indicators.

The values were not statistically pooled because the studies differed in cultivar, maturity, orchard architecture, harvesting-system configuration, sample size, damage definition, and assessment time. The table should therefore be interpreted as a structured evidence map rather than as a meta-analysis or a direct ranking of harvesting methods.

## 3. Mechanical Damage Characteristics and Physiological Consequences

### 3.1. Typical Damage Profiles of Different Harvesting Methods

Mechanical damage is not a single phenomenon. Different harvesting methods impose different mechanical loads on fruit and therefore generate distinct injury types, locations, severities, and patterns of subsequent expression [3]. Here, the characteristic combination of injuries produced by a harvesting method is termed its damage profile. A damage profile conveys more information about quality and safety consequences than a single damage rate because bruises, abrasions, cuts, and punctures differ in their implications for barrier integrity, microbial ingress, and storage deterioration [17].

According to their mechanical origin and tissue response, apple injuries can be classified as follows: (1) compression bruising, a localized compressive deformation caused by gripping, clamping, or static pressure, usually with an intact peel but ruptured subepidermal parenchyma cells; (2) impact bruising, an instantaneous injury caused by collision with a hard surface or another fruit, with tissue disruption often extending beyond the contact area [3]; (3) cuts, linear wounds in which a sharp edge breaks the peel and superficial tissues [33]; (4) punctures, localized penetrating injuries in which a pointed object passes through the peel into the flesh [34]; (5) stem-end injury, tearing at the junction between the stem and flesh during pulling or vibration [34]; and (6) abrasion, removal of epicuticular wax and epidermal cells through frictional contact [3]. Impact bruises are generally more severe than compression bruises: impact damage extends from the peel into a shear zone within the parenchyma, whereas compression damage tends to form only a thin injured layer beneath the peel [35].

The damage profiles produced by different harvesting methods are fundamentally distinct. Manual harvesting mainly involves gripping forces, localized compression, and low-height impacts, so injuries are generally mild-to-moderate compression bruises with the peel remaining intact. Vibration-based mechanical harvesting involves fruit detachment, impact with catching devices, fruit-to-fruit collision, and conveyor friction and therefore more readily produces compound injuries, including deep impact bruises, surface lacerations, punctures, and stem-end tears [8]. Damage on harvest-assist platforms occurs mainly during conveying and bin filling, when fruit may experience repeated impacts and rolling while changing direction, being elevated, and entering the bin [15]. Damage during robotic harvesting depends strongly on end-effector design and control strategy; excessive gripping force, insufficient contact area, slippage, or positioning errors can all cause localized tissue injury [31].

Damage severity is jointly affected by multiple factors. Cultivar is a primary determinant, and bruise susceptibility differs substantially among apple cultivars; ‘Jazz™’ and ‘Granny Smith’, for example, have been reported to show the lowest and highest susceptibility, respectively [36]. Maturity is also critical: as fruit mature, firmness declines and bruise susceptibility generally increases [3]. Fruit temperature can also matter. The volume of impact bruises in ‘Starkrimson Delicious’ and ‘Golden Delicious’ apples increased as fruit temperature rose from 0 to 30 °C [37], although temperature effects are cultivar-specific [19]. Peel thickness, fruit size, preharvest water management, harvest time (fruit harvested in the morning may bruise more readily than fruit harvested in the afternoon), contact-material properties, and subsequent storage conditions also affect injury development and expression [3,17]. Harvesting equipment should therefore be evaluated for specific cultivars and orchard architectures, and results from a single trial should not be generalized to all production conditions. Differences among injury types in physical barrier disruption and subsequent food-safety risk are summarized in Table 2.

### 3.2. Cell Rupture and Oxidative Stress

Apple tissue does not remain physiologically static after harvest damage. Compression and impact rupture parenchyma cells, enlarge intercellular spaces, damage membrane systems, and cause juice leakage. Enzymes, substrates, and oxygen that were previously separated by cellular compartmentalization then come into contact, initiating a cascade of physiological and biochemical reactions.

Reactive oxygen species (ROS) rapidly accumulate around wounded tissue. Mechanical injury stimulates enzymes involved in ROS metabolism, including NADPH oxidase and superoxide dismutase, increasing the production of superoxide anions and hydrogen peroxide. On the one hand, ROS act as wound signals that initiate defense and healing responses; on the other, excessive ROS promote membrane lipid peroxidation, protein oxidation, and further cell-wall degradation, creating a self-reinforcing cycle of damage expansion. Malondialdehyde content, lipoxygenase activity, and membrane permeability—direct biomarkers of lipid peroxidation and membrane integrity—are all significantly elevated in damaged fruit [38]. Apple surface tissues have been reported to exhibit diminished antioxidant capacity, intensified phenolic oxidation, and greater membrane lipid peroxidation after mechanical injury, whereas internal tissues maintain stronger jasmonate signaling and antioxidant activity.

A key feature of apple harvest injury is delayed expression. Many bruises are inconspicuous at harvest and only develop browning and tissue collapse after a short holding period or cold storage [19,39]. Hyperspectral imaging studies show that spectral characteristics in the visible–near-infrared and short-wave infrared regions continue to change for two weeks after bruising. The number of ruptured cells per unit volume of bruised tissue also increases with storage time. Consequently, visual inspection in the field or immediately after harvest cannot fully represent the actual risk associated with a harvesting method. A complete assessment should include damage expression at multiple postharvest time points, quality after storage, and shelf-life performance. Mechanical damage also triggers enhanced respiratory metabolism and ethylene biosynthesis, the detailed mechanisms and storage implications of which are discussed in Section 4.1.

### 3.3. Enzymatic Browning and Loss of Visual Quality

Enzymatic browning is the most typical and visually apparent manifestation of mechanical damage in apples. Because apples contain abundant phenolic compounds, they are highly susceptible to browning. Mechanical injury disrupts cellular compartmentalization, bringing polyphenol oxidase (PPO) into contact with phenolic substrates located in vacuoles or cell walls in the presence of oxygen. Quinones are formed and subsequently polymerize into brown pigments, commonly referred to as melanins [40]. Cell collapse is the first step in this process, and peroxidase (POX) also contributes. PPO activity differs markedly among cultivars; ‘Idared’ has been reported to exhibit the highest PPO activity and browning potential [41]. The extent of browning is jointly determined by cultivar-specific phenolic content, PPO activity, injury intensity, temperature, and time. At least 24 phenolic compounds have been detected in apples, with the peel generally containing the highest concentrations.

For the fresh-apple market, visual defects directly cause downgrading and price reductions. Bruising is commonly the leading reason for rejection during apple quality inspection [39]. Consumers interpret brown spots, depressions, abrasions, and decay as signs of reduced freshness, so market value declines substantially even when the internal flesh remains edible. One review reported that apple bruising can cause product losses of up to 50%, although typical losses are 10–25% [3]. When the total bruised area exceeds the area of a circle 0.50 inches in diameter, fruit may be downgraded from U.S. Extra Fancy to U.S. Fancy, resulting in a 20% loss of revenue [42]. In the Chinese fresh-apple supply chain, approximately 35% of production is downgraded because of cosmetic grading criteria such as bruising and lack of integrity, and approximately 17.1% is consequently regarded as food loss [43]. Commercial evaluation of mechanized harvesting must therefore treat browning expression and loss of visual grade as core outcomes. Market-value losses caused by mechanical damage affect not only the current season but also brand reputation and long-term consumer confidence. From a supply-chain perspective, visible and latent injuries introduced at harvest accumulate through grading, packaging, transport, and retail, ultimately becoming quantifiable economic losses.

## 4. Cascading Effects on Postharvest Quality and Food Safety

**Figure 2:**
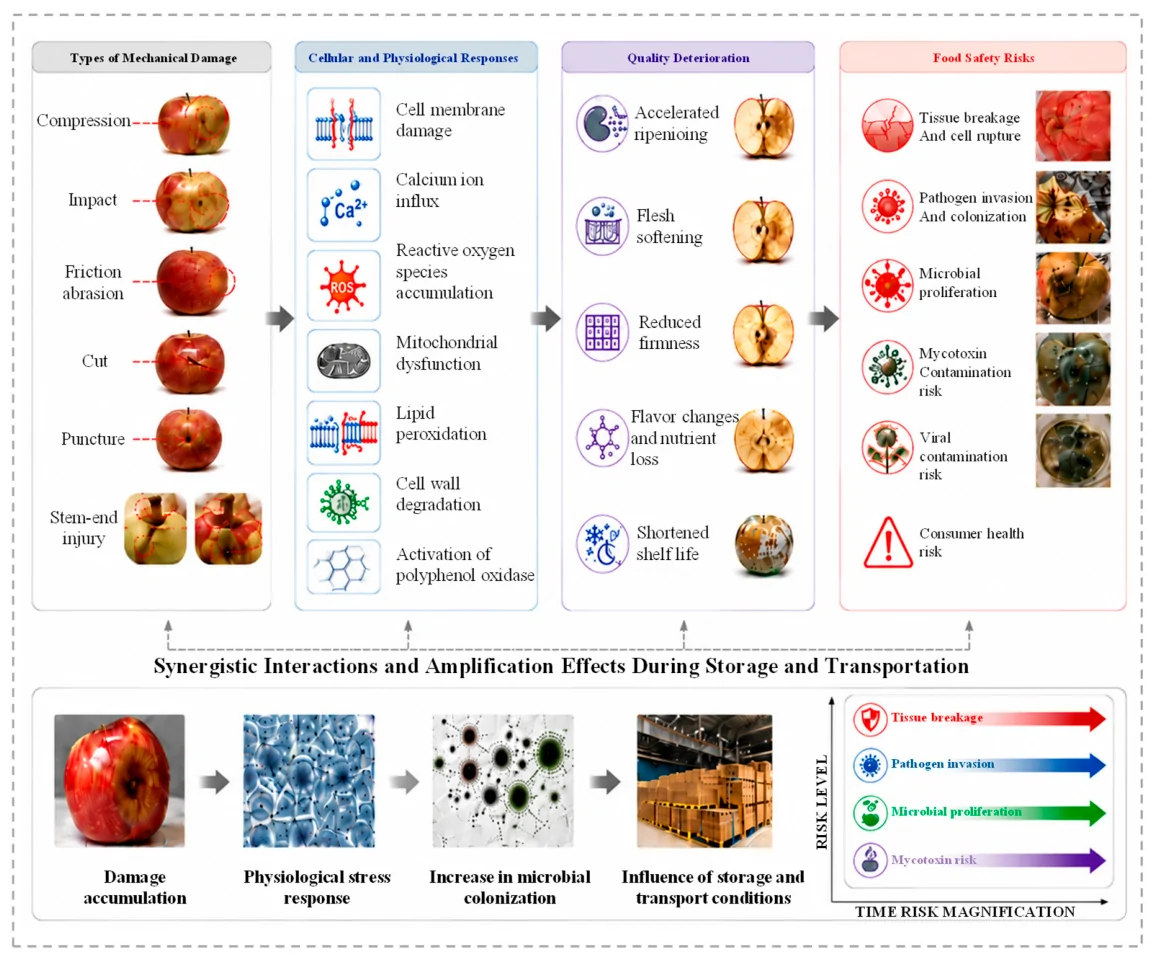
Mechanisms linking mechanical damage to quality deterioration and food-safety risks.

The consequences of harvest injury do not end when fruit leave the orchard. Rather, the cellular damage described in Section 3—membrane rupture, ROS bursts, and enzyme activation—is progressively amplified during storage and distribution, transforming microscopic tissue deterioration into macroscopic quality loss and food-safety risk. Mechanical injury ruptures cell membranes, accelerates nutrient and water loss, increases respiration, advances ripening, softens tissue, and markedly increases the probability of microbial infection, thereby impairing the quality and shelf life of fresh produce [44]. The mechanisms through which mechanical damage initiates quality deterioration and transmits risk to food safety are summarized in Figure 2. This section traces these cascading effects through the supply chain from the complementary perspectives of quality deterioration and food-safety risk.

### 4.1. Texture Softening, Flavor Changes, and Nutrient Loss

The effects of mechanical damage on apple quality first appear as coordinated deterioration in texture, flavor, and nutritional composition. Texture softening is the most prominent quality change in damaged fruit. Mechanical injury activates cell-wall-degrading enzymes, including polygalacturonase, pectin methylesterase, and cellulase, accelerating the breakdown of pectin, cellulose, and hemicellulose. Intercellular adhesion declines, and tissues lose their characteristic firmness and crispness [45,46]. Water loss and enzymatic cell-wall degradation are the two principal causes of apple softening: the former reduces cell turgor, whereas the latter directly weakens the mechanical strength of the cell wall [47]. At the same time, injury-induced increases in respiration and ethylene production further accelerate ripening and senescence. Ethylene accumulation reduces firmness, alters color, and causes aroma loss, thereby shortening shelf life [48]. The adverse effect of mechanical injury on fruit firmness has been confirmed in multiple cultivar trials. Drop impacts significantly reduce apple firmness [49], and bruised apples soften much faster than healthy fruit [50]. Water loss also accelerates softening; apples are prone to dehydration, which causes surface shriveling and deterioration of appearance and eating quality [51].

Flavor changes also warrant attention. Mechanical injury alters respiratory metabolism and disrupts the balance between synthesis and degradation of sugars, organic acids, and volatile flavor compounds [52]. Static compression increases apple respiration, rapidly reduces overall quality, and impairs flavor late in storage. At the volatile-metabolite level, fresh bruising can markedly alter the profile of apple volatile organic compounds within 0–24 h after injury [53]. Mechanical injury upregulates multiple key genes in the lipoxygenase (*LOX*) pathway as well as transcription factors such as *MYB*, *ERF*, and *WRKY* [54]. Changes in the composition and relative abundance of these compounds directly affect apple aroma and consumer acceptance.

Nutrient loss is an easily overlooked but equally important component of quality deterioration after injury. Mechanical damage causes rapid decreases in organic acids, reducing sugars, vitamin C, and total sugars in injured tissues of ‘Fuji’ apples, with larger changes at higher temperatures [50]. Damage accelerates contact between phenolic compounds and air and increases PPO and peroxidase activities, thereby promoting oxidative degradation of total phenolics and flavonoids [55]. More severe injury is associated with lower vitamin C content and water content [56]. Although consumers may not perceive these nutritional losses as readily as visual defects, they remain important from the perspective of food nutritional value [57]. Future studies of harvesting methods should therefore report not only damage rate and picking efficiency but also systematically monitor firmness, soluble solids, titratable acidity, vitamin C, total phenolics, and antioxidant capacity.

### 4.2. Barrier Disruption and the Window for Microbial Invasion

The fundamental food-safety threat posed by harvest damage is disruption of the fruit’s natural barriers. The apple peel, cuticle, and wax layer together form the first line of defense against microbial invasion [58]. Once this barrier is breached by cuts, punctures, stem-end tears, or severe impact bruises, the sugars, organic acids, and water in the flesh are exposed to environmental microorganisms, creating favorable conditions for colonization by spoilage organisms and pathogens [44].

Most postharvest pathogens cannot infect healthy tissue and typically enter fruit through dead or wounded tissue [59]. Juice exuding from damaged tissue is nutrient-rich and supports rapid microbial proliferation. Injury not only provides a physical entry route but can also facilitate infection by suppressing the fruit’s own defense responses.

Among postharvest diseases of apples, blue mold caused by *P. expansum* is one of the most important [60]. *P. expansum* is a typical necrotrophic postharvest pathogen that requires wounds—such as stem-pull injuries, punctures, bruises, or shoulder cracks—or natural openings, such as lenticels, the stem end, or the calyx cavity, to penetrate and infect fruit [61]. Damage generated during harvesting and postharvest handling provides optimal infection sites [62]. This biological characteristic creates a direct causal relationship between harvest damage and blue-mold risk: any break in the peel during harvesting can become an entry point for *P. expansum* [59]. Blue mold causes major economic losses, and *P. expansum* also produces patulin, a mycotoxin harmful to human health [63].

The type of harvest injury determines the level of microbial risk. In bruises with an intact peel, internal tissue is damaged but the cuticular barrier remains largely continuous, making direct microbial penetration difficult. The main risk is that physiological deterioration of the bruised region during storage may progress to tissue necrosis and subsequently attract pathogens. Cuts and punctures that rupture the peel directly open invasion pathways and are therefore the most hazardous injuries. Stem-end tearing deserves particular attention because the stem end is a natural weak point between the fruit and the external environment. Once this tissue is torn, microorganisms can enter the fruit core directly, and the concealed location of the injury makes it difficult to detect during grading [64]. Accordingly, food-safety assessments should assign different risk weights to different injury types.

### 4.3. Risk Amplification During Storage and Distribution

The consequences of harvest damage are often sharply amplified during storage and distribution. This amplification results from the interaction of several mechanisms.

First, as detailed in Section 4.1, the injury-induced elevation of respiration and ethylene production is sustained throughout storage, accelerating deterioration and potentially inducing ripening responses in adjacent fruit. Quality attributes of damaged fruit decline more rapidly during storage [52].

Second, the cold chain can delay but cannot eliminate damage-related risk. Rapid precooling and low-temperature storage at −1 to 1 °C effectively suppress PPO activity and microbial growth but cannot reverse tissue injury that has already occurred. Once fruit leave cold storage and enter ambient distribution or retail display, deterioration and microbial activity at injured sites rapidly resume and accelerate. As discussed in Section 6.4.1, some low-force bruises may partially recover under rapid cooling conditions [19], whereas impact bruises caused by fruit-to-fruit collisions on packing lines are more destructive and less likely to recover.

Third, delayed damage expression makes risk difficult to detect. Many bruises are inconspicuous at harvest and only develop browning and tissue collapse after several weeks of cold storage [39]. Fruit that appear acceptable during harvesting and storage intake may therefore be extensively downgraded or decayed after several months. Evaluation of mechanized harvesting technologies should not be completed on the day of harvest alone but should include simulated commercial storage and shelf-life trials. A harvesting system that produces little visible field damage but high post-storage decay, browning, or downgrading still has limited commercial value [3].

### 4.4. Patulin: From Postharvest Damage to Food-Safety Risk

Patulin is one of the most closely monitored mycotoxins in apple-related food-safety management [63]. It is a secondary metabolite produced by *P. expansum* and several other *Penicillium* and *Aspergillus* species and can cause serious adverse effects in animals and humans, particularly affecting the nervous system and potentially causing nausea and vomiting [65]. Patulin contamination has become an important determinant of the quality and safety of apples and apple juice [66].

The pathway from postharvest damage to patulin risk can be summarized as follows: mechanical injury during harvesting disrupts the peel barrier → *P. expansum* spores enter through wounds and colonize damaged tissue → the fungus grows during storage → patulin biosynthesis is activated under favorable conditions of temperature, humidity, and storage duration → the toxin accumulates in decayed tissue and may diffuse into apparently healthy adjacent tissue → damaged fruit enter the processing chain, transferring patulin into products such as juice and purée [63,66].

The critical step in this pathway is the entry route provided by tissue damage. *P. expansum* is a typical wound-invading pathogen and has difficulty infecting intact healthy fruit [59]. Peel-breaking injuries generated during harvesting, particularly cuts, punctures, and stem-end tears, therefore initiate the patulin risk chain [61]. The extent of fungal contamination on apple surfaces depends on the injury source, and patulin content has been reported to increase in the following order: mechanically damaged apples < insect-damaged apples < disease-damaged apples [66]. Apple products are commonly manufactured from stored fruit, making preharvest and postharvest strategies equally important for patulin reduction; prolonged storage, particularly beyond three months, can increase the concern [63].

Patulin risk differs among harvesting methods. Manual harvesting causes relatively few cuts and punctures and generally preserves peel integrity, so the risk is comparatively low. Vibration harvesting readily generates multiple impacts, cuts, and stem-end tears, producing the greatest proportion of peel-breaking injuries and the highest risk. Risk on harvest-assist platforms depends on conveyor cushioning and bin-filling practices. Robotic harvesting with compliant gripping and force-feedback control can reduce cutting and puncture risks, but latent bruising still requires attention. Although bruising does not immediately expose the flesh, bruised regions that progress to necrosis during storage may become secondary infection sites [59,63].

Although patulin is traditionally associated more strongly with processed products such as apple juice and applesauce, its origins often lie upstream during harvesting and storage [63]. The choice of harvesting method is therefore not merely a field-operation decision but a component of preventive food-safety control. Apples intended for processing may tolerate cosmetic defects, but requirements for excluding moldy fruit and controlling patulin are more stringent. If mechanized harvesting creates large numbers of latent injuries, damaged fruit may facilitate *Penicillium* infection and increase patulin risk during storage or bulk holding [66]. Processing use consequently does not justify ignoring harvest damage; instead, different risk thresholds should be established for different raw-material destinations.

## 5. A Conceptual Quantitative Framework for the Damage–Safety Trade-Off

The preceding sections have systematically reviewed the technological characteristics and damage profiles of four harvesting methods (Section 2), the cellular and physiological basis of mechanical injury (Section 3), and the cascading transmission of damage into quality deterioration and food-safety risks (Section 4). One conclusion emerges consistently from this evidence: not all mechanical injuries have equivalent implications for quality and safety. A superficial bruise beneath an intact peel and a puncture penetrating the peel may both be counted as damage in harvesting statistics, yet their physiological deterioration pathways, probabilities of microbial invasion, and food-safety consequences during storage are fundamentally different. Building on the dispersed evidence available in the literature, this section therefore proposes a conceptual, hypothesis-generating framework for organizing the trade-offs among efficiency, damage, quality, and safety.

It must be emphasized that the Damage Risk Index (DRI) and Harvest Suitability Score (HSS) proposed here are not empirically validated predictive models. They are conceptual tools derived from mechanistic reasoning and published evidence. The numerical weights are illustrative only and will require calibration through simulated injury, pathogen inoculation, standardized storage, toxin analysis, and cross-cultivar field validation.

### 5.1. Fragmentation of Current Evaluation Systems and the Need for an Integrated Framework

The fragmentation discussed here refers to the separation of evaluation endpoints across disciplines rather than to an absence of previous reviews. Engineering-oriented studies and reviews generally emphasize harvesting efficiency, picking success, machine reliability, and commercial feasibility. Postharvest studies commonly focus on bruise expression, firmness, browning, and storage quality, whereas food-safety studies emphasize fungal infection, decay, and mycotoxin contamination, often without treating the harvesting method as a primary experimental variable.

As summarized in Table 3, Refs. [2,10,15] provide important syntheses of harvesting equipment, automation, and commercialization. However, postharvest physiological deterioration, injury-specific food-safety consequences, and market-oriented quantitative decision support are not used as a single continuous organizing framework in these reviews. The contribution of the present review therefore lies in cross-disciplinary integration: harvesting performance is connected with injury type, delayed quality deterioration, microbial invasion, patulin risk, and supply-chain destination.

This fragmentation has two practical consequences. First, results remain difficult to compare because a reported “damage rate” may represent minor abrasion in one study but include deep bruises, cuts, or punctures in another. Second, isolated performance indicators cannot determine whether a harvesting method is suitable for premium fresh markets, long-term storage, short-term sale, or processing. The purpose of the proposed framework is therefore not to replace detailed disciplinary research, but to organize dispersed indicators into a comparable decision system.

### 5.2. Four-Dimensional Indicator System

Based on indicators repeatedly used in the literature, this review organizes the evaluation of apple harvesting methods into four dimensions: efficiency (E), damage (D), quality (Q), and safety (S). This classification does not introduce entirely new measurements; rather, it harmonizes the definitions, categories, and application contexts of existing indicators (Table 4).

During score standardization, positive indicators, such as throughput, grade retention, and raw-material acceptance rate, should be converted so that better performance receives a higher score. Negative indicators, such as DRI, decay rate, weight loss, and patulin concentration, should receive lower scores as risk increases. When sufficient data are available, min–max normalization may be used; when data are limited, categorical scoring based on expert thresholds or regulatory limits may be adopted.

Positive and negative indicators can be converted to standardized scores from 0 to 100 as follows:

(1) $${S_{i}}^{\left(+\right)}=\;100\;\times \;(x_{i}-\;x_{min})/(x_{max}-\;x_{min})$$

(2) $${S_{i}}^{\left(-\right)}=100\times (x_{max}-x_{i})/(x_{max}-x_{min})$$

where $x_{i}$ is the raw value of an indicator for a given harvesting method, $x_{min}$ and $x_{max}$ may be obtained from the same experiment, historical data, or a standardized database.

### 5.3. Differential Weighting of Injury Types and the DRI

The rationale underlying the DRI follows directly from the central findings of Section 3 and Section 4: injury types contribute differently to physical barrier disruption and the opportunity for microbial invasion and should therefore carry different weights for subsequent quality loss and food-safety risk. Bruises beneath an intact peel mainly cause browning and downgrading; cuts and punctures that break the peel directly create pathogen entry sites; and stem-end tears combine barrier disruption with poor detectability.

This review proposes the Damage Risk Index (DRI), defined as the weighted sum of the proportions of different injury types, to represent the overall damage risk of a harvesting method or fruit lot. The proposed expression is:

(3) $$DRI=\omega_{pc}P_{pc}+\omega_{st}P_{st}+\omega_{db}P_{db}+\omega_{sb}P_{sb}+\omega_{ab}P_{ab}$$

where $P_{pc}$, $P_{st}$, $P_{db}$, $P_{sb}$, and $P_{ab}$ denote the proportions of puncture/cut injuries, stem-end tears, deep bruises, shallow bruises, and abrasions, respectively; $\omega_{pc}$, $\omega_{st}$, $\omega_{db}$, $\omega_{sb}$, and $\omega_{ab}$ are the corresponding risk weights.

### 5.4. Decision-Oriented Harvest Suitability Score (HSS)

**Figure 3:**
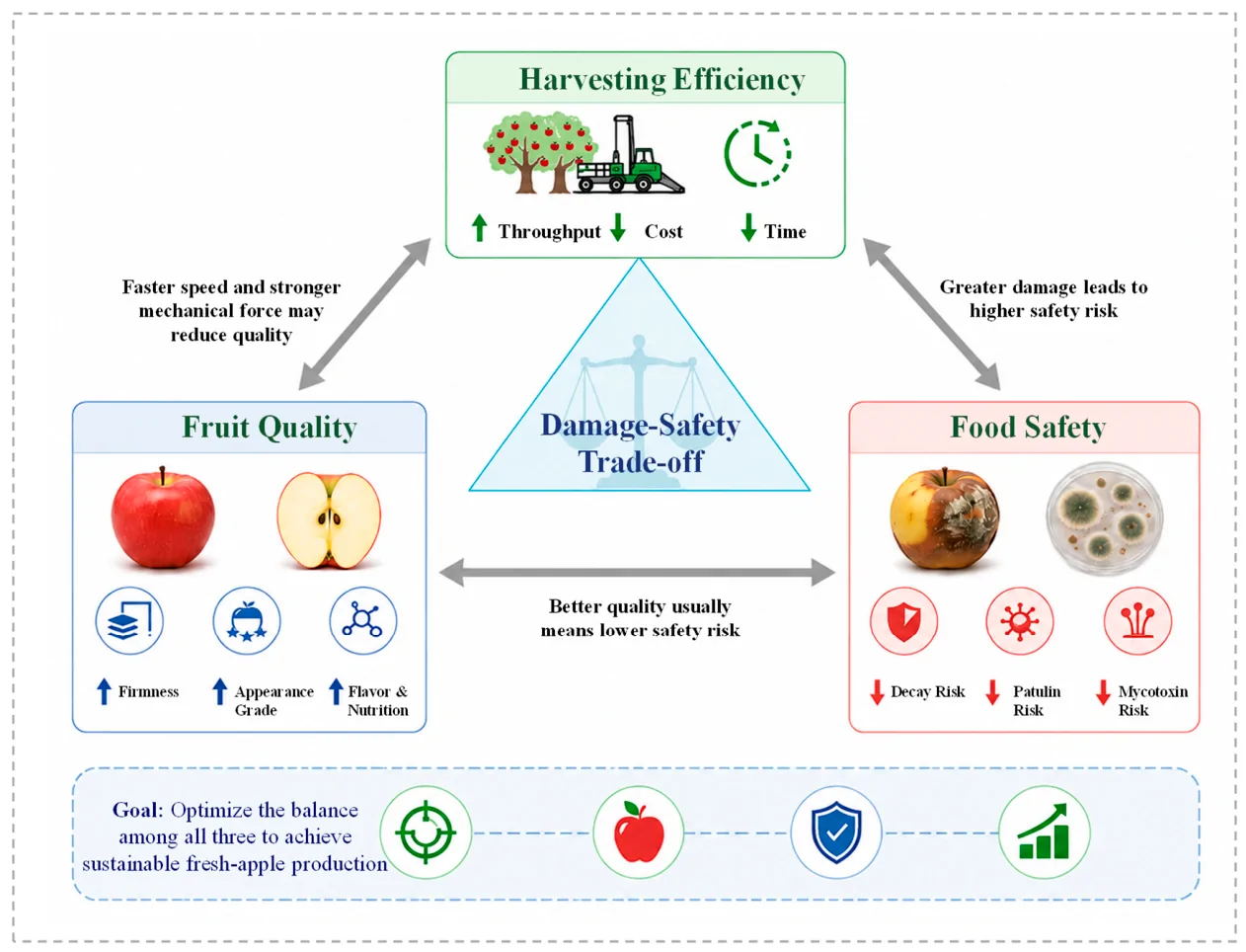
Conceptual DRI–HSS evaluation framework and its application to harvesting-method selection. Green, light blue and pink frames correspond to harvesting efficiency, fruit quality and food safety. Upward arrows rep-resent increased indicators and downward arrows represent decreased indicators. Interconnecting arrows reflect the interactions between the three dimensions.

The DRI focuses on quantifying risk at the damage level, but selection of a harvesting method must also balance efficiency, quality, and safety. We therefore further propose a Harvest Suitability Score (HSS). To illustrate how the DRI and HSS can be linked in decision-making, a conceptual evaluation framework is presented in Figure 3. The basic logic of the HSS is to convert the performance of a harvesting method in each of the four dimensions to a 0–100 score and then calculate a weighted sum using weights determined by the target market.

HSS can be expressed in general form as:

(4) $${HSS}_{k}=\lambda_{E,k}S_{E}+\lambda_{D,k}S_{D}+\lambda_{Q,k}S_{Q}+\lambda_{S,k}S_{S}$$

(5) $$\lambda_{E,k}+\lambda_{D,k}+\lambda_{Q,k}+\lambda_{S,k}=1$$

where $S_{E}$, $S_{D}$, $S_{Q}$, and $S_{S}$ are the standardized scores for efficiency, damage, quality, and safety, respectively; $\lambda_{E,k}$, $\lambda_{D,k}$, $\lambda_{Q,k}$, and $\lambda_{S,k}$ are the weighting coefficients for market scenario k. Premium fresh markets place greater emphasis on appearance, firmness, and shelf life; long-term storage should assign greater weight to latent damage and safety; and processing markets may reduce the weight of appearance but must strengthen control of moldy-fruit proportions and patulin risk.

### 5.5. Illustrative Numerical Application and Weight Sensitivity

To clarify the calculation procedure, an illustrative numerical example was constructed using hypothetical values for the four harvesting methods. This example is not an empirical validation and should not be interpreted as evidence of the actual superiority of any harvesting method. For illustration, the injury weights were set as $\omega_{pc}=1.0$, $\omega_{st}=0.8$, $\omega_{db}=0.6$, $\omega_{sb}=0.3$, and $\omega_{ab}=0.1$. These values reflect the conceptual ordering that peel-breaking injuries and concealed stem-end injuries carry greater risk than shallow bruises and abrasions, but the weights remain to be empirically calibrated.

The hypothetical injury proportions and the resulting DRI values are presented in Table 5. For example, the DRI for manual harvesting was calculated as:

(6) $$DRI_{manual}=1.0\left(0.005\right)+0.8\left(0.005\right)+0.6\left(0.020\right)+0.3\left(0.060\right)+0.1\left(0.040\right)=0.043$$

DRI was subsequently converted into the damage score $S_{D}$ using Equation (2), because a lower DRI represents better performance. Hypothetical throughput, visual-grade retention, and raw-material safety-acceptance values were standardized as positive indicators using Equation (1). Two market scenarios satisfying Equation (5) were then examined: a premium fresh-market scenario with weights $\left(\lambda_{E},\lambda_{D},\lambda_{Q},\lambda_{S}\right)=\left(0.15,0.30,0.35,0.20\right)$, and an efficiency-oriented scenario with weights $\left(0.45,0.15,0.15,0.25\right)$.

Under the premium fresh-market weights, the illustrative ranking was manual harvesting > selective robotic harvesting > harvest-assist platform > vibration harvesting. When efficiency received greater weight, the ranking changed to harvest-assist platform > selective robotic harvesting > manual harvesting > vibration harvesting. This change demonstrates that the HSS is scenario-dependent and that harvesting-method rankings can be sensitive to the selected weights. Because min–max normalization was applied only to four hypothetical alternatives, the numerical contrasts are intentionally illustrative. Empirical applications will require standardized multi-method datasets and calibrated injury and market weights.

### 5.6. Pathway for Empirical Validation of the DRI and HSS

The numerical example in Section 5.5 demonstrates the calculation procedure but does not replace empirical validation. Before practical application, both the injury weights used in the DRI and the scenario weights used in the HSS must be calibrated and tested using standardized experimental datasets.

At least four forms of validation are required before the DRI and HSS can be applied. First, injury weights should be calibrated by imposing specific simulated injuries, inoculating fruit with *P. expansum*, applying standardized storage, and measuring patulin. Second, the framework must be validated across cultivars, regions, and maturity stages because firmness, peel thickness, cuticular structure, and bruise susceptibility differ substantially among cultivars. Third, evaluation methods must be standardized; studies should report at minimum the cultivar, production location, harvest date, core temperature, precooling delay, damage-assessment time points, storage conditions, and sample size. Fourth, sensitivity analyses should determine whether changes in weights alter the ranking of harvesting methods under different market scenarios.

### 5.7. Integration with Supply-Chain Decisions

The practical value of the DRI and HSS lies in their integration into supply-chain decisions. Before harvest, growers could select manual harvesting, harvest-assist platforms, robotic harvesting, or vibration harvesting according to the target market and cultivar susceptibility. During harvest, rapid detection could be used to estimate DRI and adjust gripping force, conveyor speed, drop height, and cushioning materials. After harvest, low-DRI lots could be directed to premium fresh markets or long-term storage, medium-DRI lots to short-term sale, and high-DRI lots containing cuts or punctures to processing with intensified toxin screening. After storage and sale, decay rate, grade retention, and acceptance rate could be fed back into the traceability system to continually recalibrate the weights.

Within this architecture, the damage–safety trade-off becomes not merely an abstract concept but a decision logic that can be embedded throughout harvesting, sorting, storage, processing, and traceability.

## 6. Damage-Mitigation and Safety-Control Strategies

### 6.1. Preharvest Orchard Management and Cultivar Optimization

Apple cultivars differ substantially in susceptibility to mechanical damage [36]. Flesh firmness, cellular structure, peel thickness, wax layer, maturity, and fruit shape all affect the damage threshold [67]. The soft-textured cultivar ‘Freedom’ has been shown to be especially susceptible to surface bruising [49]. ‘Red Fuji’, which is widely grown in China, has also been characterized for bruise susceptibility under defined impact conditions [68]. Future breeding objectives should include compatibility with mechanized handling and bruise resistance in addition to yield, flavor, disease resistance, and storability [69]. In established orchards, harvesting in maturity-based batches, maintaining appropriate crop load, improving canopy openness, and reducing fruit clustering can lower the difficulty of mechanical and robotic harvesting [70].

**Figure 4:**
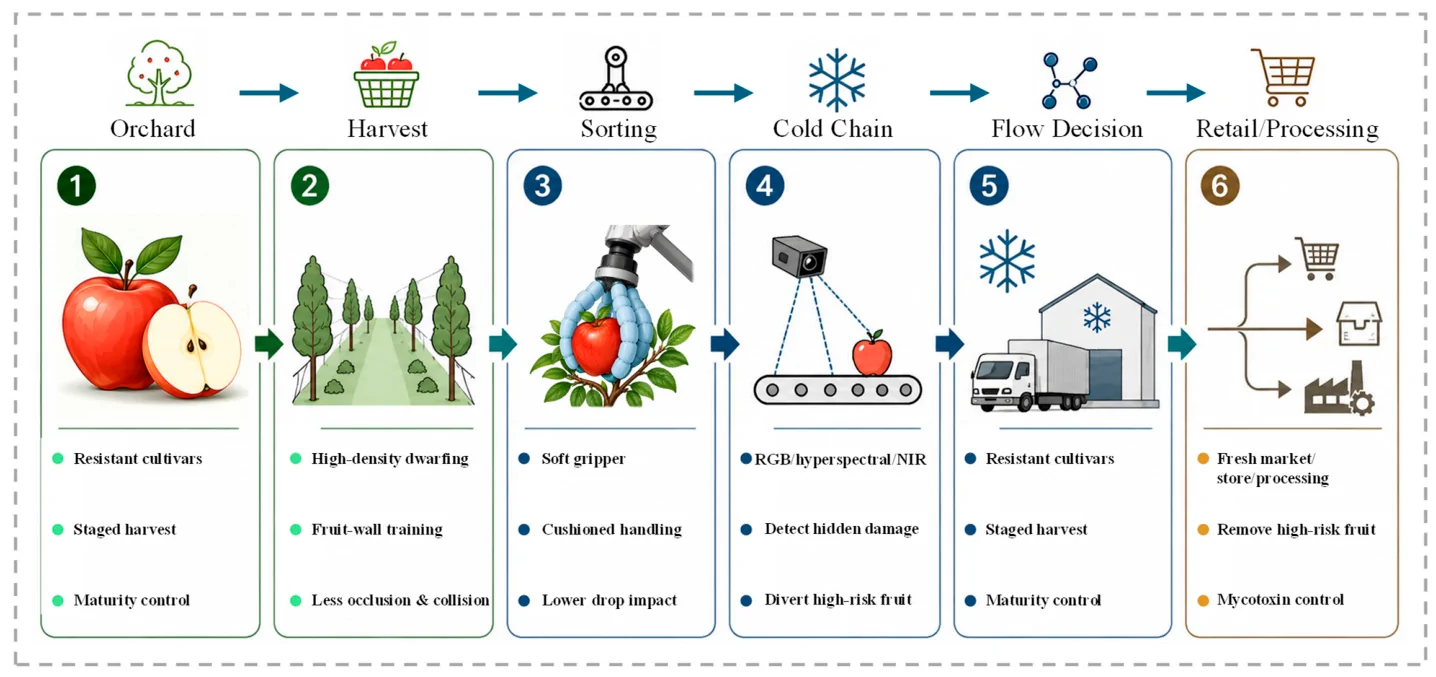
Integrated strategies for reducing harvest damage and safeguarding apple quality and food safety.

Orchard architecture is a prerequisite for successful mechanized harvesting. High-density dwarfing systems, narrow fruiting walls, and orderly canopies facilitate robotic perception, arm access, and platform operation while reducing fruit–branch collisions. Studies of the mechanical harvestability of Y-shaped and pyramid-trained ‘Empire’ and ‘Delicious’ apple trees showed that placing the catching surface close to the canopy reduced peel punctures, peel breaks, and both large and small bruises and increased the proportion of undamaged fruit [71]. Traditional tall, dense canopies increase occlusion, collision, and missed-fruit risks [72]. Approximately half of the damage during mechanical harvesting has been reported to occur near the point of fruit detachment, with the other half occurring as fruit pass through the canopy and are captured by the catching device [73]. Intelligent apple harvesting must therefore rely not only on equipment advances but also on coordinated machinery–horticulture design [74]. Integrated strategies from preharvest management to processing traceability are summarized in Figure 4.

### 6.2. Design of Low-Damage Harvesting Equipment

For vibration-based harvesting, the key objective is to reduce impact energy after fruit detachment. Damage can be mitigated by optimizing vibration frequency and amplitude, using compliant catching surfaces or air cushioning, collecting fruit in stages, reducing drop height, and minimizing conveyor collisions. The performance of an air-suspension catching mechanism has been evaluated using cultivars with different bruise susceptibilities, including ‘Granny Smith’, ‘Jazz’, ‘Honeycrisp’, ‘Fuji’, ‘Pacific Rose’, and ‘Pink Lady’ [75]. However, vibration harvesting faces a fundamental limitation for fresh-market apples: individual fruit cannot be controlled during detachment, so the risks of cuts, punctures, and multiple impacts cannot be eliminated completely [21]. In one study, only 53–72% of harvested fruit across five cultivars were undamaged; cuts and punctures were serious problems, bruising also limited quality, and apples growing within the canopy were more readily damaged than those below it [21].

For robotic harvesting, the end-effector is the central component determining damage rate [76]. Soft grippers, compliant materials, pneumatic or underactuated structures, combined suction-and-twist mechanisms, force feedback, and tactile sensors can all reduce localized pressure [77]. Soft robotic manipulators are particularly relevant in agriculture because delicate produce benefits from force distributed over a large area rather than concentrated at small contact points of rigid grippers [78]. Soft robotic systems have reduced the risks of surface bruising, rupture, tissue crushing, and plastic deformation when harvesting soft-skinned fruit such as apples, cherries, and pears [79]. An ideal end-effector should automatically adjust gripping force according to fruit size, firmness, cultivar, and maturity and detect slippage or abnormal loading in real time [80]. Excessive force causes irreversible damage and quality loss, whereas insufficient force causes slippage, dropping, and failed picks [80]. Robotic path planning should also prevent secondary collisions with branches, neighboring fruit, or machine structures [77].

### 6.3. Early Damage Detection and In-Field Sorting

#### 6.3.1. From Manual Sorting to Machine Vision

Conventional manual sorting relies on visual judgment of color, shape, and surface defects. It can identify obvious decay, lacerations, and large abrasions but has very limited ability to detect early bruising and internal tissue injury [81]. A further limitation is inconsistency, because fatigue, illumination, and operator experience cause variability in sorting thresholds [82].

Machine vision markedly improves detection consistency and speed and can be integrated into online sorting equipment. Deep-learning models are now widely used for detecting surface defects, identifying stem and calyx regions, classifying disease lesions, and screening for early bruising in apples. YOLO, Faster R-CNN, Mask R-CNN, vision transformers, and lightweight networks have all been validated in fruit-detection applications [32]. A YOLO-based apple-quality sorter can immediately classify and reject fruit with surface defects, reducing sorting time and labor costs [83].

The value of machine vision in harvest-damage control extends beyond replacing manual sorting; it can also form a closed control loop with harvesting equipment. After robotic picking, each fruit can be imaged rapidly. If a particular end-effector setting systematically increases damage, the system can immediately adjust gripping force, rotation angle, or path planning. Similarly, a vision module can be integrated into the field conveyor of a harvest-assist platform to divert damaged fruit at an early stage [84].

Standard RGB imaging nevertheless has inherent limitations for detecting latent bruises. Red or dark-skinned cultivars can mask slight browning; calyx and stem cavities, natural color patches, and wax reflections can cause false detections [85]; and variable field illumination, shadows, fruit curvature, and motion blur further reduce model robustness. RGB images alone are therefore unlikely to meet the early-damage detection requirements of high-grade fresh-market apples.

#### 6.3.2. Spectral Imaging and Multisensor Fusion

Hyperspectral, near-infrared, and short-wave infrared imaging can capture subtle changes in tissue structure, water status, pigment composition, and chemical constituents and therefore offer unique advantages for detecting early bruises. Hyperspectral imaging (HSI), which integrates spectral and spatial information, has transformed the nondestructive detection of fruit defects [86]. After injury, cell rupture alters light scattering, while changes in water status and browning-related reactions shift characteristic spectra. Classification can therefore use selected wavelengths, band ratios, principal component analysis, partial least-squares discriminant analysis, support vector machines, or deep-learning models [86]. Combining near-infrared hyperspectral imaging (NIR-HSI) with geometric influence correction has enabled enhanced early-bruise detection across apple cultivars with large size variation [87]. The FP-YOLOv5 model achieved a 95% recognition rate for apple bruises at 130 frames per second [32].

The main barriers to engineering application of spectral imaging are cost, speed, and system complexity. Hyperspectral systems provide rich information but generate high-dimensional data, acquire images relatively slowly, are expensive, and require strict illumination and calibration. Commercial online sorting is more likely to require multispectral systems using a small number of critical wavelengths for high-speed detection [88]. Future research should move from laboratory detectability to online deployability through wavelength selection, model compression, transfer learning, and edge computing.

A single sensing technology usually captures only one dimension of fruit condition, whereas harvest damage involves structural disruption, color change, chemical-composition shifts, and potential microbial activity. Multisensor fusion could therefore substantially improve both detection and risk prediction [89]. RGB imaging can screen external defects, short-wave infrared imaging can detect latent bruises, and gas or volatile sensors can provide early warnings of decay [90]; decision-level fusion can then generate an integrated risk classification.

#### 6.3.3. From Defect Recognition to Risk Prediction

Future detection systems should do more than determine whether damage is present. They should also predict whether a particular injury will develop into a food-safety risk during storage or distribution. This transition requires upgrading binary defect recognition into an integrated decision system combining injury-type classification, severity quantification, and shelf-life risk prediction [86].

A shallow bruise beneath an intact peel and a puncture penetrating the peel are fundamentally different from a microbiological perspective. The former may remain acceptable for short-term sale, whereas the latter can carry substantial decay and toxin-production risk even when its initial area is small [82].

Risk-prediction models should integrate multiple inputs, including mechanical harvesting parameters (gripping force, impact velocity, and contact material), image-derived features, cultivar, maturity, and anticipated storage temperature, humidity, and distribution time. Machine-learning models trained on historical lot data could predict the probability that an individual fruit will be downgraded during future storage and automatically recommend the most appropriate destination: premium fresh market, standard fresh market, short-term sale, processing, or rejection. Under this architecture, harvest-damage detection becomes an intelligent supply-chain decision node rather than an isolated quality-control step [91].

An integrated harvesting–sorting–precooling system represents a system-level implementation of this concept. If high-risk damaged fruit can be identified in the field or immediately after harvest and diverted to processing, short-term sale, or rejection, the probability that they enter long-term storage or premium fresh-market channels can be markedly reduced [92]. The application scenarios, advantages, and constraints of different nondestructive detection technologies are summarized in Table 6. This strategy directly reduces quality loss and also provides upstream protection against subsequent patulin risk.

### 6.4. Cold Chain, Hygiene, and Control of Processing Raw Materials

The risks associated with harvest damage do not end when fruit leave the orchard. On the contrary, adverse effects on quality and safety are often amplified during subsequent cooling, packaging, transportation, and retail [93]. Cold-chain management and coordinated supply-chain control constitute the final defense in the damage–safety pathway. Their purpose is not to repair existing injury, but to suppress its expression and spread through systematic temperature control, hygienic management, and lot segregation [93]. Stage-specific coordinated measures and evaluation indicators are presented in Table 7.

#### 6.4.1. Cold-Chain Management

As discussed in Section 4.1, damage-induced elevation of respiration and ethylene production makes injured lots more dependent on an uninterrupted cold chain than sound fruit. Damaged lots therefore depend more strongly on an uninterrupted cold chain than sound lots.

Rapid precooling is the first line of control after injury [19]. If field heat and respiratory heat are not removed promptly, PPO activity around wounds and microbial proliferation both increase substantially. When simulated harvest force was low and fruit were immediately cooled to 1 °C, most bruises recovered visibly within several weeks; recovery was much less pronounced when bruising force was high or cooling was delayed [19]. Lots at high risk of damage should therefore be precooled within 2–4 h after harvest, reducing core temperature to 0–4 °C. Stable low temperatures of −1 to 1 °C and relative humidity of 90–95% can delay senescence, reduce water loss, and suppress fungal growth [94]. Controlled-atmosphere storage with O_2_ reduced to 1–3% and CO_2_ increased to 1–3% can further reduce respiration and ethylene action [95], although it cannot reverse pre-existing tissue injury [95]. Apples stored under ultra-low-oxygen conditions (2% CO_2_ and 2% O_2_) have shown lower bruise susceptibility and decay and better quality than fruit held under conventional controlled atmospheres [94].

The cold chain delays rather than eliminates damage-related risk. Once fruit leave refrigerated conditions and enter ambient distribution or retail display, tissue deterioration and microbial activity at injured sites rapidly resume [66]. Bruises caused at harvest may partially recover during storage, whereas impact bruises caused by fruit-to-fruit contact on packing lines are more destructive and less likely to recover [19]. High-damage lots should therefore be stored for shorter periods, inspected more frequently after removal from storage, and kept separate from premium fresh-market fruit. Secondary impacts and compression during packaging and transportation must also be controlled through cushioning liners, layered stacking, and fewer handling operations [93].

#### 6.4.2. Upstream Control Strategies for the Fresh-Apple Supply Chain

For fresh-market apples, harvest-damage control should begin during harvest-system design rather than relying on corrective sorting at the packinghouse [82]. Producers should first define acceptable damage thresholds according to cultivar characteristics, maturity, and target market:

- Premium fresh-market and long-term-storage lots: use manual harvesting or low-damage harvest-assist platforms, strictly limit drop height and picking-bag fill depth, and avoid emptying fruit from excessive heights. A compression force of only 22.2 N can produce detectable bruising, although most low-force bruises may visibly recover within several weeks if fruit are immediately cooled to 1 °C [19].
- Standard fresh-market lots: platform-based harvesting and in-field conveying may be used to a greater extent, but conveyor speed, bend radius, and bin-filling height must be controlled, with fruit placed gently in layers. In-field sorting can separate grades during harvesting, allowing high-quality apples to be sold at a premium for fresh consumption while lower-grade fruit are directed to juice, fresh-cut products, or jam [82].
- Robot-harvested lots: before commercial-scale adoption, plot-scale validation trials should compare 24 h damage expression, post-cold-storage decay, and grade retention under different end-effector settings. Adoption decisions should not be based solely on picking success.

Fresh-apple supply chains also require lot-based management [96]. Fruit harvested by different methods, from different orchard blocks, or at different maturity stages should not be mixed in the same long-term storage lot [97]. Fruit with higher damage risk should be diverted to short-term sale or processing, whereas low-damage lots should be prioritized for premium markets, export, or controlled-atmosphere storage [98]. This diversion strategy follows the same logic as risk-based food-safety management: fruit should not all be handled under one standard but directed according to their risk level.

#### 6.4.3. Safety Control of Apples Intended for Processing

Processing apples are often assumed to tolerate greater mechanical damage, but this assumption requires qualification from a food-safety perspective. Patulin, the principal mycotoxin of concern in apples, is produced mainly by *P. expansum* during storage [66]. Cosmetic defects have relatively little effect on the appearance of juice or purée, but moldy fruit, punctures, stem-end tears, and prolonged holding of damaged fruit markedly increase microbial load and patulin risk. Patulin production is strongly temperature-dependent: low temperatures generally prevent fungal growth and mycotoxin formation, whereas high or fluctuating temperatures promote toxin accumulation in infected fruit [99]. Controlled-atmosphere storage may also suppress patulin accumulation [66]. Apples intended for juice, purée, jam, or infant products should not be held for prolonged periods at ambient temperature after harvest; damaged lots should enter washing, sorting, and processing as soon as possible [100]. When immediate processing is not possible, they should be temporarily stored below 4 °C with adequate ventilation to prevent rapid mold development at wound sites [101].

1. Processing facilities can establish a dedicated control procedure for mechanically harvested raw material:
2. Record the harvesting method, harvest date, and holding duration at intake and establish lot-level traceability records [96];
3. Use coordinated manual inspection and machine vision to remove moldy, cracked, and severely punctured fruit [81];
4. Conduct targeted patulin testing for high-risk lots, such as vibration-harvested fruit, fruit harvested in rain, or fruit held for excessive periods [102];
5. Determine raw-material use according to test results: compliant lots may enter ordinary juice or fermentation streams, whereas noncompliant lots should be downgraded, diverted, or discarded [103];
6. Trace the causes of noncompliant lots and feed the results back to adjustments in harvester settings and orchard management.

The purpose of this procedure is to convert uncertain damage risk from mechanized harvesting into a structured, monitorable, and feedback-driven quality-control problem. The internationally recognized HACCP system has been adopted as a theoretical basis for more effectively reducing patulin in apple juice concentrate [102].

#### 6.4.4. Digital Traceability and Closed-Loop Supply-Chain Optimization

The distinctive advantage of intelligent harvesting systems lies not only in precise robotic manipulation and visual perception but also in their inherent capacity for data generation [104]. Each picking action can record the time, location (GPS coordinates or orchard-block identifier), cultivar, estimated maturity, equipment parameters (gripping force, suction pressure, and end-effector type), pick success or failure, and rapid postharvest inspection results [96]. Linking these records with subsequent storage quality, sorting outcomes, and market feedback creates a harvest database oriented toward food quality [105].

The value of accumulating these data is evident at several levels:

Continuous optimization: companies can identify orchard blocks, cultivars, canopy positions, or equipment settings associated with elevated damage and then adjust harvesting strategies and orchard management accordingly [106].

Risk warning: if an unusually high proportion of latent bruising is detected in a lot on the harvest day, the system can recommend shorter storage, more frequent inspection, or diversion to short-term sales [98]. If a robotic end-effector repeatedly causes excessive damage on a particular cultivar, the system can recommend replacing the compliant material or adjusting gripping force. If processing raw material originates from a high-damage mechanical-harvest lot, the system can automatically intensify patulin sampling [107].

Research support: conventional postharvest studies are often limited by uncontrolled differences among lots and environments, whereas digital harvest records provide finer explanatory variables and covariates [108]. Blockchain offers an immutable and transparent ledger with potential advantages for fresh-food quality traceability and dynamic monitoring [104]. Standardized open datasets that combine harvesting parameters, fruit images, damage grades, storage quality, and safety indicators would substantially support interdisciplinary model development and improve algorithm generalizability [104].

## 7. International Standards, Regulations, and Upstream Food-Safety Control

The food-science significance of apple harvest damage is also reflected in its relationship with quality standards and safety regulations. Commercial grades for fresh-market apples generally consider shape, coloration, size, surface defects, pests and diseases, and flesh integrity [109]. Codex, UNECE, USDA, and Chinese standards for fresh apples all identify decay, wounds, pronounced abrasion, severe bruising, and defects affecting eating quality as important limiting factors [110,111,112]. Although terminology and tolerances differ, the common principle is that flesh should remain sound and defects should not materially impair appearance, quality, storability, or package presentation. Harvest damage is therefore not simply a field-operation issue; it directly affects grading, trade, and retail performance [3].

From a food-safety perspective, patulin regulations and control guidance further reinforce the upstream importance of damage prevention. The U.S. Food and Drug Administration has established an action level of 50 μg/kg (50 ppb) for patulin in apple juice and related products [113]. Juice HACCP guidance notes that patulin formation is influenced by several factors, particularly whether raw apples include windfalls, whether fruit show visible damage at harvest—such as bird or insect injury, mold, or decay—and whether bruising occurs before storage or during handling. European Union legislation establishes different maximum levels for fruit juices, solid apple products, and apple products intended for infants and young children [114]. JECFA established a provisional maximum tolerable daily intake of 0.4 μg/kg body weight per day [115]. The Codex Code of Practice for the Prevention and Reduction in Patulin Contamination in Apple Juice and Apple Juice Ingredients (CXC 50-2003) explicitly emphasizes avoiding rotten, moldy, or severely damaged fruit [116]. Although these provisions primarily regulate processed products, the origin of risk often lies upstream during harvest and storage [63].

Risk-control logic differs between fresh-market and processing apples. Fresh fruit require intact appearance, stable eating quality, and long shelf life, whereas processing apples may tolerate some cosmetic defects but require stricter exclusion of moldy fruit and control of mycotoxins. Large numbers of latent bruises from mechanized harvesting may not immediately affect juice yield but can promote *Penicillium* infection and increase patulin risk during storage or holding [66]. Processing use therefore does not mean that harvest damage can be ignored; destination-specific risk thresholds are required [63]. Recommendations for linking harvest-damage parameters with existing quality and safety standards and HACCP systems are provided in Table 8.

Standards and regulations also indicate that harvesting trials should report damage using more comparable definitions. Many studies provide only a damage rate or bruise rate without specifying injury area, depth, peel rupture, assessment time, or whether damage was allowed to develop during storage [17]. When studies use different thresholds, reported rates have limited comparability [3]. Future work should classify injuries into four categories: minor superficial defects that do not affect marketability; visible bruises that reduce grade; wounds that compromise the peel barrier; and severe injuries likely to create food-safety risk. These categories should be linked to the relevant commercial-grade requirements.

In commercial supply chains, selection of a harvesting method should be integrated with the company’s quality-management system and HACCP approach [117]. For fresh-market apples, key control points include gentle handling during picking, cushioning of field containers, in-field sorting, rapid precooling, cold-chain stability, and reinspection before sale. For apple juice and other apple products, key control points include raw-material acceptance, removal of decayed fruit, washing and sorting, patulin testing, and lot traceability [102]. Mechanized or intelligent harvesting systems that automatically record picking time, orchard block, equipment parameters, and damage-detection results could support a quality-traceability chain from the orchard to processing or retail [104].

## 8. Research Gaps and Future Directions

First, systematic comparative trials across harvesting methods, cultivars, and storage periods are urgently needed. Ideally, manual harvesting, harvest-assist platforms, vibration harvesting, and robotic harvesting should be compared within the same orchard or closely matched orchards. Damage expression, grade, firmness, weight loss, browning, decay, and safety indicators should be evaluated on the harvest day, after precooling, during mid-storage, at the end of storage, and during shelf life. Only such designs can reveal the true effects of harvesting method on food outcomes.

Second, quantitative relationships between injury type and food-safety risk should be established. Compression, impact, abrasion, cutting, puncture, and stem-end injuries can be simulated under controlled conditions and combined with natural storage or *P. expansum* inoculation to measure lesion development, fungal populations, and patulin production. These data would provide an experimental basis for injury-risk weights, sorting thresholds, and decisions on raw-material destination.

Third, robotic harvesting research should expand from engineering feasibility to food-quality evaluation. Most studies emphasize recognition accuracy, positioning error, picking success, and cycle time per fruit, but few systematically assess postharvest quality and safety. A higher-level performance metric should be successful picking without subsequent food-quality degradation, rather than merely whether a fruit was detached from the tree.

Fourth, cultivar, training system, and equipment should be optimized jointly. Mechanical-load tolerance differs among cultivars, and tree architecture strongly affects robotic accessibility and collision risk. Compatibility with mechanization should be treated as a shared objective in the development of new cultivars, production systems, and equipment rather than addressed through passive adaptation to traditional orchards.

Fifth, integrated models are needed for industry decision-making. Such models should incorporate labor cost, equipment cost, picking speed, damage rate, grade loss, storage loss, toxin-testing cost, and market price within a single framework. For producers, the most valuable question is not whether a technology appears advanced in an experiment, but whether it increases net returns and reduces risk under the specific conditions of their orchard, cultivar, and market.

Sixth, standardized datasets and open validation should be strengthened. Apple-damage detection models are affected by cultivar, illumination, equipment, postharvest interval, and imaging conditions, and the absence of cross-regional datasets limits generalization. Open datasets should include multiple cultivars, injury types, postharvest time points, imaging conditions, and subsequent storage outcomes to support the transition of nondestructive detection and robotic harvesting algorithms from the laboratory to commercial use.

## 9. Conclusions

The transition from manual to mechanized and intelligent apple harvesting is being driven by labor shortages, rising costs, and orchard modernization. For fresh-market apples, however, harvesting efficiency cannot be evaluated separately from food quality and safety. Mechanical injury disrupts the peel and cellular structure, induces oxidative stress and enzymatic browning, accelerates softening and nutrient loss, promotes decay, and can increase patulin risk. Each harvesting method has a distinct damage profile. Manual harvesting remains the quality benchmark for fresh fruit; vibration-based mechanical harvesting is efficient but poses greater risks for fresh-market use; harvest-assist platforms are a practical transitional option; and selective robotic harvesting offers low-damage potential but still requires advances in speed, reliability, cost, and food-quality evaluation.

The damage–safety trade-off framework developed in this review indicates that future evaluations of harvesting technology should simultaneously include efficiency, damage, postharvest quality, food safety, and economic performance. Commercial development should not seek a single technology for every scenario, but should adopt differentiated strategies according to market destination, cultivar susceptibility, orchard architecture, and cold-chain capacity. Premium fresh markets should prioritize control of latent bruising and grade retention; processing markets should focus on excluding moldy fruit and controlling patulin; and robotic and harvest-assist systems should be integrated with early nondestructive detection, in-field sorting, and digital traceability.

Ultimately, the goal of intelligent apple harvesting is not simply to detach fruit faster, but to deliver safe, high-quality, traceable food more consistently at an acceptable cost. Deep interdisciplinary research spanning food science, agricultural engineering, postharvest physiology, and food safety is therefore essential for high-quality mechanization and intelligent upgrading of the apple industry.

## Figures and Tables

**Table 1:** Representative quantitative findings reported in studies of apple harvesting methods and damage control.

Category	Reference	Reported Quantitative Finding	Main Implication	Important Limitation
Manual harvesting	[4]	Harvesting accounted for approximately 20–35% of total apple-production costs	Manual harvesting has a substantial labor and cost burden	Economic endpoint; no directly comparable damage percentage
Vibration harvesting	[20]	Mechanical harvesting of ‘Brown Snout’ cider apples required approximately 22% of the labor cost of hand harvesting	Strong labor-saving potential for processing apples	Cider cultivar and processing destination
Vibration harvesting	[8,22]	Approximately 85% of fruit met the highest USDA grade under optimized conditions; approximately 15% were downgraded	Damage remains a barrier to premium fresh-market use	Grade-based endpoint rather than injury-specific assessment
Harvest-assist platform	[24]	Positive economic benefits were consistently obtained above 7.6 ha, whereas negative benefits were obtained below 4.2 ha under the evaluated yield scenarios	Economic feasibility depends strongly on orchard scale and yield	Economic result rather than direct damage rate
Selective robotic harvesting	[28,29]	Approximately 80% first-attempt picking success, 91% highest-grade retention, and 2.4–4.9% bruise incidence	Relatively low damage is possible, but reliability remains important	Field conditions and system configuration differed
Selective robotic harvesting	[30]	All evaluated fruit met Extra Fancy grade; picking success was 82.4% in young orchards and 65.2% in older orchards	Orchard architecture strongly affects performance	Different orchard structures and test conditions
Postharvest damage control	[19]	A compression force of 22.2 N produced detectable bruising; many low-force bruises visibly recovered after immediate cooling to 1 °C	Damage expression depends on force and cooling delay	Simulated injury rather than complete harvesting systems
Bruise detection	[32]	The FP-YOLOv5 model achieved 95% recognition at 130 frames s^−1^	High-speed detection may support online or in-field sorting	Recognition accuracy does not directly represent storage or safety risk

This table indicates that useful quantitative evidence is available, but the endpoints are heterogeneous. Some studies report labor cost or picking success, whereas others report grade retention, bruise incidence, or detection accuracy. This lack of common endpoints prevents direct statistical comparison and supports the need for the standardized E–D–Q–S indicator system proposed in Section 5.

**Table 2:** Mechanical injury types, degree of barrier disruption, and food-safety significance.

Food-Safety Significance	Main Quality Consequences	Detectability	Degree of Barrier Disruption	Tissue Characteristics	Injury Type
Direct safety risk is low, but extensive abrasion can increase weight loss and opportunities for surface microbial colonization.	Visual downgrading and increased water loss	Low	Low to moderate	Damage to the wax layer and epidermal cells.	Abrasion
Short-term direct risk is low, but necrotic tissue may develop during prolonged storage.	Enzymatic browning, indentation, and downgrading	High	Low	Peel generally remains intact; subepidermal parenchyma cells rupture under compression.	Shallow compression bruise
Expansion of necrosis during storage can create a secondary site for pathogen invasion.	Browning, softening, loss of firmness, and shortened shelf life	High	Moderate	Impact energy penetrates deeply into the flesh, causing a broad area of tissue collapse.	Deep impact bruise
Can create a hidden entry site and should be recorded separately as a high-risk injury.	Stem-end decay and internal deterioration	High	High	Tearing at the stem–flesh junction; the injury is concealed.	Stem-end tear
Provides a direct entry route for pathogens such as *P. expansum* and forms an upstream node in the patulin risk chain.	Localized browning, juice leakage, and rapid decay	Moderate	Highest	The peel and flesh are directly penetrated by a sharp object or branch.	Cut/puncture

**Table 3:** Point-by-point comparison of previous apple-harvesting reviews and the present review.

Comparison Item	Ref. [15]	Ref. [10]	Ref. [2]	Present Review
Main focus	Development and commercialization of mechanical apple harvesting	Progress and bottlenecks of fresh-market mechanical harvesting	Automated harvesting equipment, perception, localization, and robotics	Damage–safety trade-offs from harvesting to food outcomes
Main harvesting technologies	Semi-automatic harvesters, robots, and harvest-assist platforms	Shake-and-catch systems, robots, and harvest-assist platforms	Vision systems, robotic harvesters, end-effectors, and harvest-assist technologies	Manual, vibration-based mechanical, harvest-assist, and selective robotic harvesting
Main evaluation endpoints	Bruising, efficiency, cost, and commercialization	Detachment, catching, bruising, reliability, economics, and adoption	Recognition, localization, picking performance, automation, and equipment development	Efficiency, injury type, postharvest quality, safety, and market suitability
Treatment of mechanical damage	Mainly considered as a commercialization barrier	Mainly considered as a technical bottleneck, particularly in catching operations	Mainly considered as an equipment-performance outcome	Classified into compression, impact, abrasion, cuts, punctures, and stem-end injuries
Postharvest physiology	Not a central organizing framework	Not a central organizing framework	Not a central organizing framework	Dedicated discussion of ROS, browning, softening, respiration, and nutrient loss
Food-safety pathway	Not a central organizing framework	Not a central organizing framework	Not a central organizing framework	Explicit connection to *P. expansum*, decay, and patulin risk
Quantitative decision support	No integrated E–D–Q–S framework	No integrated E–D–Q–S framework	No integrated E–D–Q–S framework	DRI, HSS, market-specific weighting, and sensitivity analysis
Supply-chain application	Mainly technology development and adoption	Mainly technology development and adoption	Includes potential links to sorting and bagging	Harvesting, sorting, precooling, storage, processing, HACCP, and traceability

This comparison concerns the primary scope and organizing framework of each review. It does not imply that previous reviews contain no individual observations related to fruit quality or damage.

**Table 4:** Four-dimensional indicator system for evaluating apple harvesting methods.

Purpose	Recommended Measurement Time	Preferred Direction	Representative Indicators	Dimension
Evaluate operational efficiency and cost savings.	During harvesting	Higher is better; a lower missed-fruit rate is better.	kg h^−1^, fruit s^−1^, picking success, missed-fruit rate, and proportion of labor saved	Efficiency (E)
Evaluate the initial risk introduced by the harvesting method.	Immediately after harvest, 24 h, 7 d, and at storage removal	Lower is better.	Total damage rate, bruise area/depth, cut and puncture rate, stem-end injury rate, and latent-damage rate	Damage (D)
Evaluate actual fresh-market quality outcomes.	After precooling, during storage, after storage removal, and during shelf life	Most indicators: higher is better; weight loss and decay: lower is better.	Visual-grade retention, firmness, soluble solids, titratable acidity, weight loss, browning index, decay rate, and shelf life	Quality (Q)
Evaluate food-safety risk and regulatory compliance.	After storage, before processing, and during product testing	Risk indicators should be lower; acceptance rate should be higher.	Pathogen infection rate, mold incidence, patulin detection frequency and concentration, and raw-material acceptance rate	Safety (S)

**Table 5:** Illustrative calculation of the DRI and HSS using hypothetical data for four harvesting methods.

Harvesting Method	Hypothetical Injury Proportions, %	DRI	$S_{E}$	$S_{D}$	$S_{Q}$	$S_{S}$	Premium-Market HSS	Efficiency-Oriented HSS
Manual harvesting	0.5/0.5/2/6/4	0.0174	0.0	100.0	100.0	100.0	85.0	55.0
Vibration harvesting	8/6/15/20/12	0.1066	100.0	0.0	0.0	0.0	15.0	45.0
Harvest-assist platform	1/1/5/10/8	0.0344	50.0	80.9	80.6	75.0	75.0	65.5
Selective harvesting robot	0.5/0.5/4/7/5	0.0234	26.7	93.3	90.3	89.3	81.5	61.9

Injury proportions are listed in the order puncture/cut injury, stem-end tear, deep bruise, shallow bruise, and abrasion. Hypothetical throughput values were 100, 400, 250, and 180 $kg\cdot h^{-1}$; hypothetical visual-grade retention rates were 96%, 65%, 90%, and 93%; and hypothetical safety-acceptance rates were 98%, 70%, 91%, and 95% for manual, vibration-based, harvest-assist, and robotic harvesting, respectively. All input values and weights are hypothetical and are used only to illustrate the calculation procedure.

**Table 6:** Application of nondestructive detection and multisource sensing technologies to harvest-damage risk identification.

Suitable Stage	Limitations	Advantages	Detectable Targets	Detection Technology
In-field sorting and packing-line grading	Poor detection of early latent bruises and internal damage	Low cost, easy deployment, and suitability for high-speed sorting	Visible bruises, abrasions, abnormal color, and shape defects	RGB machine vision
Rapid field screening and pre-storage inspection	Higher equipment cost and demanding model transfer	Sensitive to latent bruises and suitable for early detection	Subsurface bruises, water-status changes, and tissue-structure abnormalities	NIR/SWIR imaging
Research validation and targeted inspection of critical lots	Large data volume and difficulty in real-time engineering deployment	High information content and potential for severity prediction	Early bruises, disease risk, and quality differences	Hyperspectral imaging
Cold-store monitoring and shelf-life warning	Specificity is affected by environment and cultivar	Potential for risk warning during storage	Early decay volatiles and abnormal respiratory metabolism	Gas/volatile sensors
Real-time robotic harvesting control	Requires deep integration with robotic control systems	Direct feedback for end-effector control	Gripping force, contact pressure, and slippage	Force/tactile sensors
Closed-loop harvesting–sorting–storage systems	Requires cross-cultivar and cross-scenario datasets	Supports progression from defect recognition to risk prediction	Injury type, severity, decay, and safety risk	Multisource fusion

**Table 7:** Coordinated supply-chain strategy for controlling apple harvest damage.

Stage	Core Objective	Key Measures	Evaluation Indicators
Preharvest	Reduce the difficulty of mechanized operations	Harvest cultivars and maturity classes separately; manage canopy openness; plan the harvest window	Maturity uniformity, fruit accessibility, and predicted damage risk
Harvest	Reduce initial mechanical damage	Control gripping force, drop height, and contact materials; optimize platform conveying and robotic paths	Immediate damage rate, latent-damage rate, and picking efficiency
In-field sorting	Prevent high-risk fruit from entering long supply chains	Use machine vision or spectral detection; divert fruit by damage grade	Removal accuracy, false-rejection rate, and diversion proportion
Storage and transport	Suppress amplification of damage	Rapid precooling, stable low temperature, fewer secondary impacts, and lot-based management	Decay rate, firmness retention, and grade retention
Processing or retail	Safeguard safety and consumer acceptance	Raw-material acceptance, toxin testing, shelf-life monitoring, and traceability feedback	Patulin compliance rate, complaint rate, and economic loss

**Table 8:** Recommendations for linking apple harvest damage with quality and safety standards.

Standard or Control Target	Primary Focus	Relationship to Harvest Damage	Suggested Research Indicators
Fresh-apple grade standards	Appearance, sound flesh, surface defects, decay, pests, and diseases	Bruises, abrasions, cuts, and punctures cause downgrading	Visible-defect rate, Extra Fancy/first-grade retention, and flesh integrity
Patulin regulations	Mycotoxin limits in apple juice and apple products	Damaged and moldy fruit are major contamination sources	Proportion of decayed fruit, patulin testing, and raw-material rejection rate
HACCP and good operating practices	Identification of critical control points and preventive risk management	Harvesting, sorting, storage, and pre-processing can all become risk nodes	Harvest-lot traceability, equipment hygiene, and sorting effectiveness
Export and retail quality requirements	Package presentation, shelf life, and consumer acceptance	Latent bruises may become visible during transportation and sale	Post-simulated-storage damage expression and shelf-life compliance

## Data Availability

No new data were created or analyzed in this study. Data sharing is not applicable to this article.
